# Supplementary material for: The Association between Party Horn Use and Respiratory Function in Patients with Dementia: An Experimental Study
Source: Medicina (Kaunas). 2023 Jan 10;59(1):134. doi: 10.3390/medicina59010134 (PMC9866139; doi:10.3390/medicina59010134)
Supplement: Supplementary file 1 [file medicina-59-00134-s001.zip › Table_S1.pdf]

SUPPLEMENTARY INFORMATION

Table S1. Assessment of cognitive function

The nine items below are evaluated using three responses: “Can” or “No” = 2 points, “Sometimes” = 1 point, and “Cannot” or “Yes” = 0 point.

1. Communication of resolve.
2. Comprehension of everyday daily duty.
3. Tell birth date.
4. Short-term memory.
5. Tell one’s own name.
6. Comprehension of the current season.
7. Comprehension of location.
8. Loitering.
9. Cannot get back after going out.

Higher scores indicate higher cognitive function.
