# Supplementary material for: The Association between Party Horn Use and Respiratory Function in Patients with Dementia: An Experimental Study
Source: Medicina (Kaunas). 2023 Jan 10;59(1):134. doi: 10.3390/medicina59010134 (PMC9866139; doi:10.3390/medicina59010134)
Supplement: Supplementary file 1 [file medicina-59-00134-s001.zip › Table_S2.pdf]

SUPPLEMENTARY INFORMATION

Table S2. Assessment of mental and behavioral disorders

The 14 items below are evaluated based on three responses: “No” = 2 points, “Sometimes” = 1 point, and “Yes” = 0 point.

1. Mischievous thinking
2. Confabulation
3. Unstable emotion
4. Day–night reversal
5. Talk about the same topics
6. Let out a big noise
7. Resistance to nursing care
8. Restless
9. Collectomania
10. Destroy stuff or clothes
11. Serious memory loss
12. Soliloquizing or laughing to oneself
13. Selfish behavior
14. Unfocused talk

Lower scores indicate more severe mental and behavioral disorders.
