# Supplementary material for: The Association between Party Horn Use and Respiratory Function in Patients with Dementia: An Experimental Study
Source: Medicina (Kaunas). 2023 Jan 10;59(1):134. doi: 10.3390/medicina59010134 (PMC9866139; doi:10.3390/medicina59010134)
Supplement: Supplementary file 1 [file medicina-59-00134-s001.zip › Table_S3.pdf]

SUPPLEMENTARY INFORMATION

Table S3. Assessment of eating-related behavioral problems

The below 18 items are evaluated based on two responses: "Yes" = 0 point and "No" = 2 points.
1. Try to eat with hands.
2. Try to put hands into the dish.
3. It is hard to use cutlery, such as a spoon or fork.
4. It is hard to choose meals on some of the dishes.
5. It is hard to concentrate on meals and tendency to leave the table during meals.
6. Try to touch others' meals.
7. Try to play with food.
8. Try to put a wet hand towel or napkin into the mouth.
9. It is hard to maintain a high arousal level.
10. Processed foods at all times.
11. Accumulated foods in the buccal cavity.
12. Try not to open the mouth while eating.
13. Stuff foods too swiftly into the mouth.
14. Try to eat foods by sucking.
15. Refusal to eat.
16. Try to have a droopy posture.
17. Try to bend the neck backward.
18. Try to talk when the mouth is full.

Higher scores indicate less severe eating-related behavioral problems.
