# Supplementary material for: The Association between Party Horn Use and Respiratory Function in Patients with Dementia: An Experimental Study
Source: Medicina (Kaunas). 2023 Jan 10;59(1):134. doi: 10.3390/medicina59010134 (PMC9866139; doi:10.3390/medicina59010134)
Supplement: Supplementary file 1 [file medicina-59-00134-s001.zip › Table_S4.pdf]

SUPPLEMENTARY INFORMATION

Table S4. Assessment of dietary modifications

The four items below are evaluated based on five responses: "not applicable" = 4 points, "1 item concerned" = 3 points, "2 items concerned" = 2 points, "3 items concerned" = 1 point, and "all items concerned" = 0 point.
1. Need fabricating help, such as shredding foods.
2. Need to thicken foods.
3. Need to use a smaller spoon size.
4. Need support to eat.

Higher scores indicate less need for dietary modification.
